# Clinical predictors of TRAb decline after total thyroidectomy in patients with Graves’ disease

**DOI:** 10.1530/ETJ-25-0284

**Published:** 2026-01-07

**Authors:** Takahiro Sasaki, Minoru Kihara, Makoto Fujishima, Hiroo Masuoka, Takuya Higashiyama, Yasuhiro Ito, Naoyoshi Onoda, Akihiro Miya, Akira Miyauchi, Takashi Akamizu

**Affiliations:** ^1^ Departments of Head and Neck Surgery, Kuma Hospital, Kobe, Japan; ^2^ Departments of Surgery, Kuma Hospital, Kobe, Japan; ^3^ Departments of Internal Medicine, Kuma Hospital, Kobe, Japan

**Keywords:** Graves’ disease, thyrotropin receptor antibody (TRAb), total thyroidectomy, immunological remission, TRAb kinetics, predictive factors, Graves’orbitopathy

## Abstract

**Objective:**

Thyrotropin receptor antibodies (TRAbs) are central to Graves’ disease management, but their long-term kinetics after total thyroidectomy is poorly defined. We aimed to describe long-term TRAb trajectories and identify predictors of decline using clinically relevant thresholds.

**Methods:**

TRAb levels were measured serially in 1,516 patients after total thyroidectomy. Primary outcomes were time to TRAb < 10 and <2 IU/L, corresponding to a pragmatic fetal-risk threshold and assay negativity, respectively. Clinical factors (age, sex, body mass index, smoking, preoperative TRAb, and thyroid weight) were evaluated using Kaplan–Meier analyses and multivariable logistic regression.

**Results:**

TRAb levels declined rapidly, with median values falling below 10 IU/L within 2 years. Among patients with preoperative TRAb ≥ 10 IU/L, 84.8% reached <10 IU/L and 60.1% reached <2 IU/L within 5 years. In multivariable models, younger age, lower preoperative TRAb, and smaller thyroid weight independently predicted earlier decline, whereas sex, body mass index, and smoking did not.

**Conclusions:**

In this large post-operative cohort, younger age emerged as an independent predictor of accelerated TRAb decline after total thyroidectomy. Total thyroidectomy yields sustained TRAb decline and may promote immunological remission, particularly in younger patients, by removing the antigenic source before the establishment of long-lived plasma cells. These findings highlight age as a key determinant of TRAb kinetics and provide timelines for counselling and follow-up. Surgery may be especially valuable when a timely TRAb decline is required, such as in younger patients planning pregnancy or those with orbitopathy or antithyroid drug refractoriness.

## Introduction

Graves’ disease (GD) is an autoimmune disorder characterised by the production of stimulating thyrotropin receptor antibodies (TRAbs), which drive hyperthyroidism by activating thyroid follicular cells (1). TRAb serves as both a marker of disease activity and a predictor of clinical outcomes. Persistent elevation is linked to relapse after antithyroid drug withdrawal (2) and complications, including Graves’ orbitopathy (GO) (3, 4) and neonatal thyrotoxicosis (5, 6).

Although antithyroid drugs and radioactive iodine (RAI) are commonly used, total thyroidectomy provides rapid and definitive control of hyperthyroidism (2, 7). It may also accelerate immunological remission by removing the antigenic source. Surgery is indicated for medication intolerance or poor response, large goitre, and the need for early remission (e.g. before pregnancy) (8). These indications are consistent with the 2018 European Thyroid Association guidelines on the management of Graves’ hyperthyroidism, which emphasise thyroidectomy as an appropriate option in these clinical scenarios (9).

TRAb levels decline after total thyroidectomy; however, the kinetics and determinants of post-operative decline remain unclear (2, 10). The therapeutic paradigm for GD has remained largely unchanged for decades, relying on ATDs, radioiodine, or surgery (7). Few large-scale studies have examined how age, sex, BMI, thyroid weight, and smoking status affect TRAb decline. The aim of this study was to clarify the long-term kinetics of TRAb following total thyroidectomy in a large cohort of patients with GD and to identify clinical factors associated with early TRAb decline, thereby improving the understanding of the immunological effects of surgery. Beyond describing how rapidly TRAb falls, clarifying how many patients remain above clinically relevant thresholds over long time horizons addresses an unmet need for counselling for pregnancy and orbitopathy. Using Kaplan–Meier and multivariable models, we quantified long-term TRAb trajectories after total thyroidectomy and identified predictors of earlier decline (age, baseline TRAb, and thyroid weight).

## Materials and methods

This retrospective observational study included 1,516 patients with GD who underwent total thyroidectomy at our institution between 2009 and 2022. The inclusion criteria were at least 1 year of post-operative follow-up and the availability of pre-operative and post-operative TRAb data. Patients with a history of prior RAI therapy or thyroid surgery, and those with coexisting thyroid cancer or parathyroid disease, were excluded. All total thyroidectomies were performed by high-volume thyroid specialists using a standardised technique with bilateral RLN/parathyroid preservation and no intentional remnants. GD was diagnosed as typical thyrotoxicosis with diffuse goitre and/or increased radionuclide uptake and/or positive TRAb; orbitopathy and Doppler hypervascularity supported this diagnosis. Patients with pre-operative TRAb levels <2.0 IU/L were retained if clinical and scintigraphic features supported GD, reflecting real-world surgical practice.

TRAb was measured using the same third-generation, M22-based ECLIA (Roche, Switzerland) throughout (2009–2022); third-generation assays outperform earlier methods in sensitivity/specificity and reproducibility, supporting longitudinal comparability (11, 12).

The manufacturer’s reference interval for negative results at our institution was <2.0 IU/L during the entire study period. The upper detection limit of the assay was 400 IU/L; values exceeding this threshold were recorded as 400 IU/L. Most patients visited the outpatient clinic at least every 6 months post-operatively. Blood samples were collected at each visit, and TRAb levels were measured to monitor disease activity.

Primary endpoints were reaching TRAb levels <2.0 IU/L (assay-based negativity) and <10 IU/L post-operatively. We pre-specified two thresholds: <2.0 IU/L (assay-based negativity) and <10 IU/L. Although guidelines typically recommend a screening threshold of three times the upper limit of normal for fetal monitoring, we selected <10 IU/L as a clinically actionable cut-off because values above this level strongly and specifically predict overt neonatal thyrotoxicosis requiring treatment (13, 14). Therefore, this threshold serves as a robust practical target for avoiding clinically significant adverse outcomes. The proportion of patients reaching these thresholds was calculated at each follow-up. The Kaplan–Meier analyses were used to estimate the time to TRAb levels <10 IU/L, stratified by

- Sex (male vs female).
- Age at surgery (<40 vs ≥40 years).
- BMI (<25 vs ≥25 kg/m^2^).
- Pre-operative TRAb (<100 vs ≥100 IU/L).
- Resected thyroid weight (<200 vs ≥200 g).
- Smoking status (never vs current smoker).

For thyroid weight, we pre-specified <200 vs ≥200 g; ≥200 g was chosen *a priori* as a pragmatic threshold for ‘very large’ goitres in our surgical practice. The results were directionally similar when thyroid weight was modelled as a continuous variable (data not shown). In our cohort, resected thyroid weight showed a strong correlation with pre-operative ultrasound-estimated volume (*r* = 0.955), supporting its validity in clinical risk stratification. Smoking status was self-reported at baseline. We classified the patients as current or never smokers, and excluded past smokers to reduce misclassification. Log-rank tests were used to assess group differences.

Because our primary endpoints were binary (reaching TRAb levels <10 IU/L within 1 year and within 3 years post-operatively), we used multivariable logistic regression analyses to identify independent predictors. Both models included sex, age, BMI, smoking status, pre-operative TRAb levels, and thyroid weight. The correlation between pre-operative TRAb levels and resected thyroid weights was assessed using Spearman’s correlation and a scatter plot. In the sensitivity analysis, age, BMI, pre-operative TRAb levels, and thyroid weight were also modelled as continuous variables in the multivariable logistic regression (Supplementary Tables 1A and 1B (see the section on Supplementary materials given at the end of the article)).

All statistical analyses were performed using StatFlex (version 7.0; Artec, Japan). Statistical significance was set at *P* < 0.05.

This retrospective study was approved by the Ethics Committee of Kuma Hospital (Approval No. 20250612-3) and conducted in accordance with the Declaration of Helsinki, as revised in 2013, and the institutional guidelines. The requirement for written informed consent was waived by the Ethics Committee owing to the retrospective design, and an opt-out consent process was implemented via public notification on the hospital website.

## Results

### Patient characteristics

A total of 1,516 patients who underwent total thyroidectomy for GD were included in the analysis (Table 1).

**Table 1:** Clinical characteristics and surgical data of patients undergoing total thyroidectomy for Graves’ disease (*n* = 1,516).

Variable	Value
Male/female (female %)	268/1,248 (82.3%)
Age at surgery (years)*	37 (8–82)
Post-operative follow-up period (months)*	57 (12–188)
Pre-operative TRAb (IU/L)*	20.3 (0.3–400)
Weight of resected thyroid (g)*	97.6 (7.3–832.4)
Operative time (min)*	112 (49–320)
Intraoperative blood loss (g)*	35 (2–1,273)
Post-operative hospital stay (days)*	5 (2–38)

* Median (range).

### Post-operative TRAb trends

**Figure 1:**
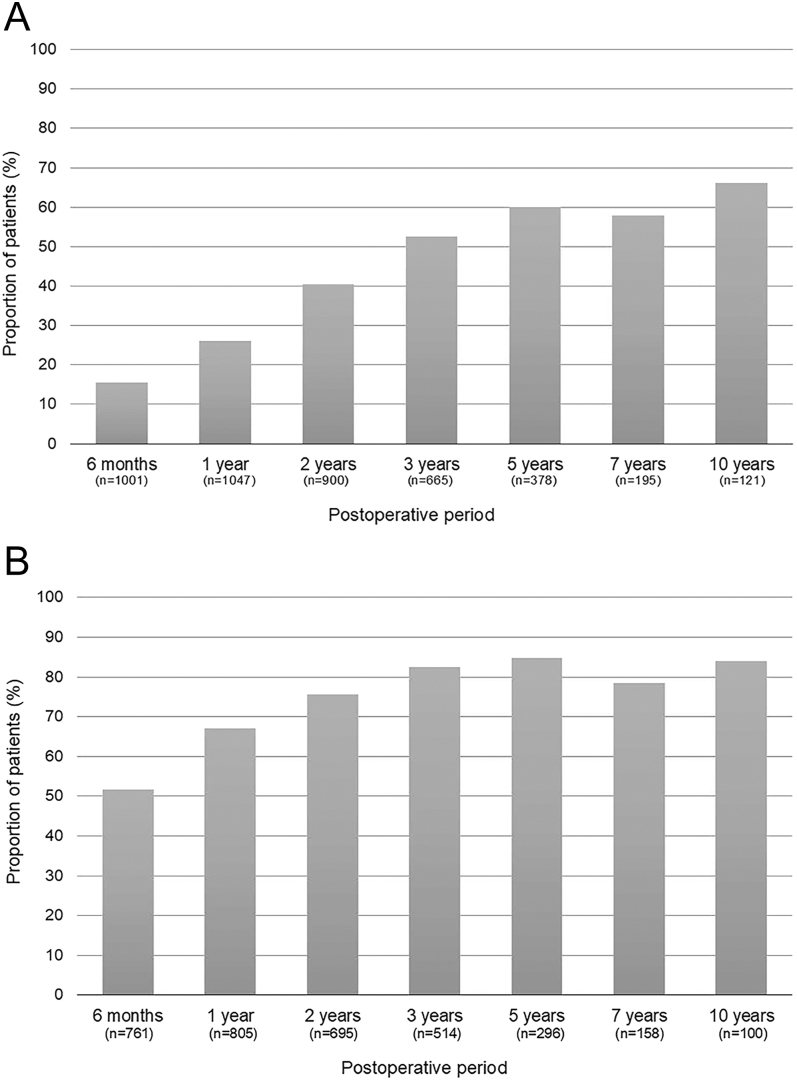
Cumulative proportion of patients reaching TRAb normalisation after surgery. (A) Cumulative proportion of patients reaching a TRAb level <2 IU/L in those with pre-operative TRAb ≥ 2 IU/L (*n* = 1,416). The proportion steadily increased, exceeding 60% at 5 years. (B) Cumulative proportion of patients reaching a TRAb level <10 IU/L in those with a pre-operative TRAb level ≥10 IU/L (*n* = 1,045). Most patients achieved this threshold within 3–5 years, with nearly 85% reaching a TRAb level of <10 IU/L by year 5.

The mean and median TRAb levels declined progressively over time, with a sharp initial decrease in the first 6 months, followed by a gradual, sustained reduction. The longitudinal trends are shown in Supplementary Fig. 1. Of the 1,416 patients with pre-operative TRAb levels ≥2 IU/L, 52.6% reached TRAb levels <2 IU/L within 3 years and 60.1% within 5 years (Fig. 1A). Of the 1,045 patients with pre-operative TRAb levels ≥10 IU/L, 82.5% reached TRAb levels <10 IU/L within 3 years and 84.8% within 5 years (Fig. 1B). Given the clinical importance of pregnancy planning, we performed a stratified analysis of female patients with pre-operative TRAb levels ≥10 IU/L according to the age group (Table 2). The decline in TRAb was particularly favourable in women of reproductive age. In the 20–29 age group, 60.2 and 86.8% of patients achieved TRAb levels <10 IU/L at 1 and 3 years post-operatively, respectively. Similarly, in the 30–39 age group, these proportions were 63.1 and 88.2%, respectively. As shown in Table 2, these rates of normalization tended to be higher than those observed in the older age groups (40–49 and ≥50 years). While the median TRAb levels fell below 10 IU/L by ∼2 years, the levels in a clinically meaningful subset, particularly those with very high baseline titres, remained ≥10 IU/L at 5 years and were still measurable at 10 years (∼40%), indicating heterogeneity and a potential plateau.

**Table 2:** Proportion of female patients with pre-operative TRAb levels ≥10 IU/L achieving TRAb levels <10 IU/L after total thyroidectomy, stratified by age. Data are presented as percentage (number of patients with TRAb < 10 IU/L/total number of patients with available data). Included are female patients with pre-operative TRAb levels ≥10 IU/L.

Age group (years)	1 year post-op	2 years post-op	3 years post-op
20–29	60.2% (65/108)	71.7% (71/99)	86.8% (66/76)
30–39	63.1% (99/157)	75.6% (102/135)	88.2% (90/102)
40–49	55.7% (54/97)	65.1% (54/83)	72.7% (48/66)
≥50	50.9% (54/106)	59.1% (52/88)	69.0% (49/71)

### Stratified TRAb dynamics by pre-operative levels

The baseline TRAb strata were 2–10 (*n* = 371), 10–50 (*n* = 650), 50–100 (*n* = 131), and ≥100 IU/L (*n* = 264). These categories were chosen to reflect clinically intuitive severity bands while maintaining adequate sample sizes in each stratum.

**Figure 2:**
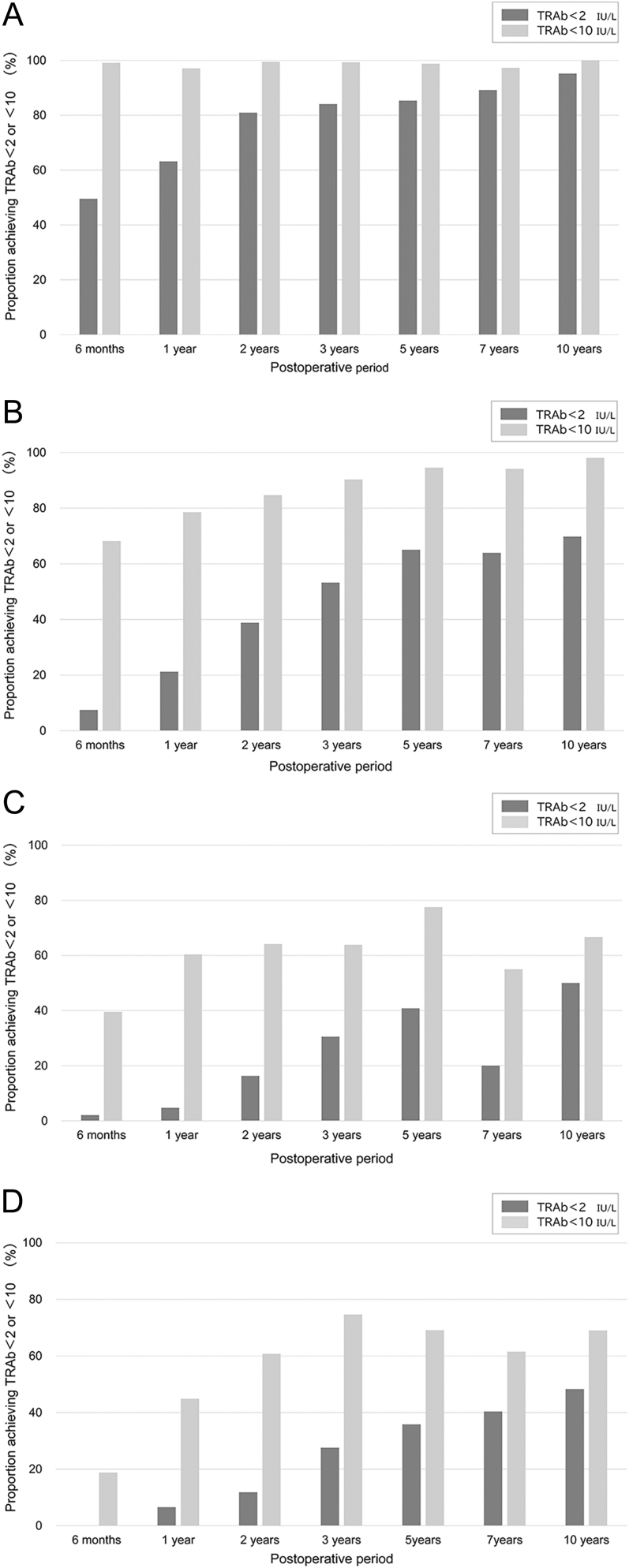
Proportion of patients with TRAb < 2 IU/L and <10 IU/L at each post-operative time point, stratified by pre-operative TRAb levels. The bar graphs show the percentage of patients reaching TRAb levels of <10 IU/L (light grey) and <2 IU/L (dark grey) at each post-operative time point. Patients were stratified according to their pre-operative TRAb levels: (A) 2–10 IU/L (*n* = 371), (B) 10–50 IU/L (*n* = 650), (C) 50–100 IU/L (*n* = 131), and (D) >100 IU/L (*n* = 264). Overall, patients with higher initial TRAb levels showed slower and delayed normalisation compared to those with lower preoperative values.

**Figure 3:**
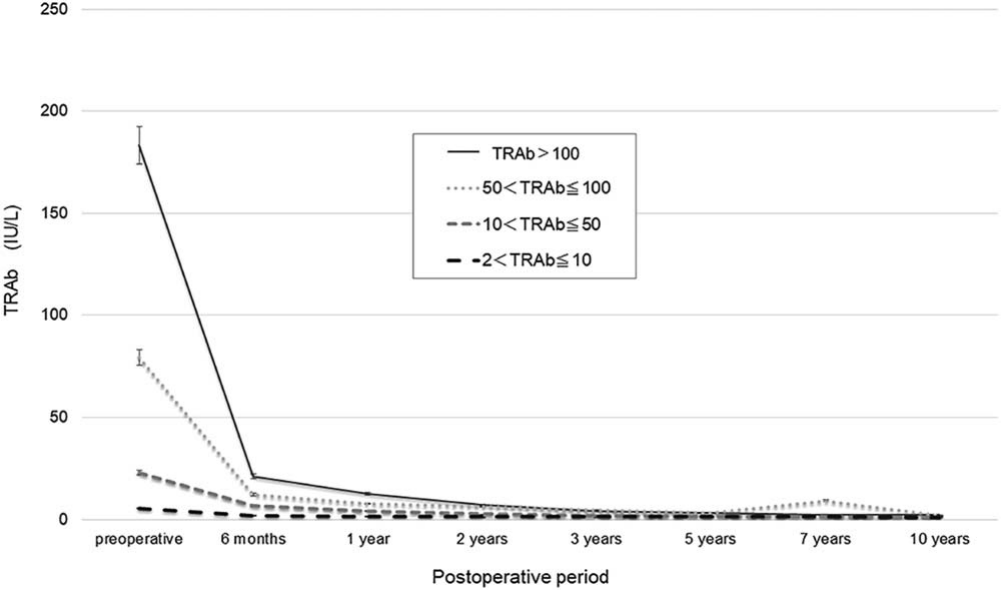
Median TRAb levels over time by pre-operative TRAb category. The graph shows the longitudinal changes in the median TRAb levels stratified by the pre-operative TRAb groups. Despite the baseline differences, all groups exhibited a sharp initial decline within 6 months, with the median levels falling below 10 IU/L by 2 years post-operatively.

Figures 2A, B, C, D illustrate that patients with higher pre-operative TRAb levels showed a slower, more delayed decline than those with lower values. Among the 30 patients with extremely high pre-operative TRAb levels (≥400 IU/L), none reached TRAb levels <10 IU/L within 1 year. However, 33.3, 50.0, and 64.3% of the patients reached this threshold by years 2, 3, and 5, respectively. Figure 3 shows that despite different starting titres, all groups showed a sharp decline within 6 months, and the median TRAb levels dropped below 10 IU/L by 2 years in each group.

### Clinical factors associated with TRAb decline (Kaplan–Meier analysis)

**Figure 4:**
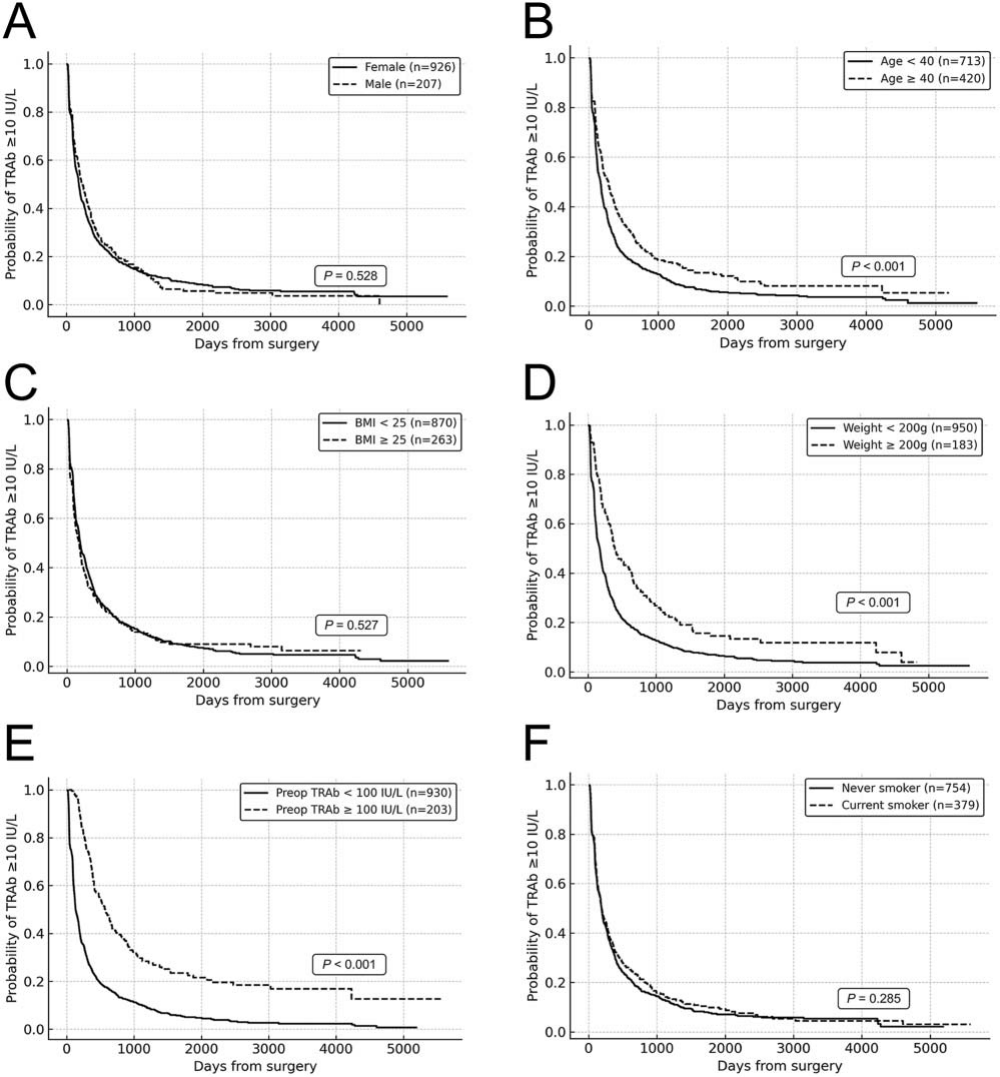
Time to achieve TRAb < 10 IU/L stratified by clinical variables. The Kaplan–Meier curves illustrate the time to reach a TRAb level <10 IU/L after total thyroidectomy, stratified by the following clinical variables: (A) sex: no significant difference was found between males and females (*P* = 0.528). (B) Age: patients aged <40 years reached normalisation significantly earlier than those aged ≥40 years (*P* < 0.001). (C) BMI: no significant difference was observed between BMI < 25 and ≥25 (*P* = 0.527). (D) Thyroid weight: a thyroid weight ≥200 g was associated with delayed TRAb decline (*P* < 0.001). (E) Pre-operative TRAb level: patients with TRAb ≥ 100 IU/L had significantly slower normalisation (*P* < 0.001). (F) Smoking status: no significant difference was noted between never and current smokers (*P* = 0.285).

Patients aged <40 years reached TRAb levels <10 IU/L earlier (*P* < 0.001; Fig. 4B), whereas those with a thyroid weight ≥200 g and baseline TRAb levels ≥100 IU/L had a delayed decline (both *P* < 0.001; Fig. 4D and E). Sex and BMI showed no differences (both *P* > 0.5; Fig. 4A and C), and smoking was not significant (*P* = 0.285; Fig. 4F).

### Correlation between TRAb and thyroid weight

A significant positive correlation was observed between pre-operative TRAb levels and resected thyroid weight (Spearman’s *ρ* = 0.35, *P* < 0.001), indicating that higher TRAb titres were linked to a larger thyroid volume (Supplementary Fig. 2).

### Predictive models for TRAb decline

Within 1 year, age ≥40 years (OR = 0.60, 95% CI: 0.46–0.79), baseline TRAb levels ≥100 IU/L (0.18, 0.13–0.26), and thyroid weight ≥200 g (0.59, 0.41–0.86) predicted delayed TRAb decline; the same was true within 3 years (0.79, 0.69–0.90; 0.43, 0.36–0.53; 0.73, 0.60–0.89) (Table 3A and B). Sex, BMI, and smoking showed no significant associations. In sensitivity analyses modelling age, BMI, preoperative TRAb, and thyroid weight as continuous variables, the results were generally consistent: older age, higher preoperative TRAb levels, and larger thyroid weight remained significantly associated with delayed achievement of TRAb < 10 IU/L at both 1 and 3 years, whereas BMI was not (Supplementary Tables 1A and 1B).

**Table 3:** Multivariable logistic regression analysis identifying predictors of reaching TRAb level <10 IU/L within A) 1 year and B) 3 years after total thyroidectomy. This table presents the odds ratios (ORs), 95% confidence intervals (CIs), and *P* values for clinical variables associated with reaching TRAb level <10 IU/L within 1 year or 3 years after surgery. The TRAb threshold of 10 IU/L was used as a surrogate marker for clinical immunological remission. Female sex, age ≥40 years, BMI ≥ 25, pre-operative TRAb ≥ 100 IU/L, thyroid weight ≥200 g, and current smoking status were included as explanatory variables. A lower OR indicates a lower likelihood of reaching TRAb level <10 IU/L within 1 year or 3 years.

Variable	OR	95% CI	*P* value
A) Within 1 year			
Female sex	0.97	0.68–1.39	0.862
Age ≥ 40	0.60	0.46–0.79	<0.001
BMI ≥ 25	1.19	0.86–1.64	0.288
TRAb ≥ 100 IU/L	0.18	0.13–0.26	<0.001
Thyroid ≥ 200 g	0.59	0.41–0.86	0.005
Current smoker	0.73	0.44–1.21	0.221
B) Within 3 years			
Female sex	1.02	0.86–1.21	0.834
Age ≥40	0.79	0.69–0.90	<0.001
BMI ≥ 25	1.08	0.93–1.26	0.308
TRAb ≥ 100 IU/L	0.43	0.36–0.53	<0.001
Thyroid ≥ 200 g	0.73	0.60–0.89	0.002
Current smoker	1.02	0.89–1.18	0.765

## Discussion

In this large surgical cohort, the TRAb levels declined rapidly after total thyroidectomy, and most patients reached <10 IU/L within 3–5 years; however, a clinically meaningful subset remained above the thresholds at late follow-up, indicating a long tail and potential plateau. At 5 years, 60.1% (baseline TRAb levels ≥2 IU/L) had reached <2 IU/L and 84.8% (baseline TRAb levels ≥10 IU/L) had reached <10 IU/L. Younger age, lower baseline TRAb level, and lower thyroid weight independently predicted earlier decline.

### Influence of pre-operative TRAb levels on post-operative trends

Higher pre-operative TRAb levels were associated with delayed post-operative decline. Previous studies have linked elevated baseline TRAb levels to prolonged antibody persistence (10, 15). TRAb, which is produced by autoreactive B cells, has a long half-life, crosses the placenta, and is retained through neonatal Fc receptor recycling (16). Persistent elevation may reflect high antibody loads and continued autoantibody production after surgery, possibly due to extrathyroidal TSH receptor expression on orbital fibroblasts or bone marrow-derived fibrocytes (17). Only approximately half of the patients with very high pre-operative TRAb levels (>100 IU/L) reached TRAb levels <10 IU/L at 1 year post-operatively. Nevertheless, all subgroups showed an initial TRAb decline within 6 months, and the median levels fell below 10 IU/L by 2 years, indicating that remission is achievable even in high-risk cases, given sufficient follow-up.

### Correlation between TRAb and thyroid weight

Supplementary Fig. 2 shows a moderate positive correlation between pre-operative TRAb levels and thyroid gland weight, supporting prior reports that larger thyroids are associated with increased autoimmune activity (17, 18). Antigenic stimulation from an enlarged gland may contribute to high TRAb levels, with thyroid weight potentially serving as both a marker and a modulator of autoimmune dynamics.

### Age and other clinical predictors

Smoking status, previously implicated in GD (19), had no significant impact on TRAb decline, even when the analysis was limited to active smokers. This contrasts with the previous reports suggesting that smoking delays TRAb reduction (10) and may partly reflect the lack of longitudinal smoking data in our study. This discrepancy may be partly attributable to the lack of longitudinal smoking data in this study. We relied on self-reported status at baseline and could not verify peri-operative abstinence or post-operative relapse; thus, any null association should be interpreted with caution. Moreover, because younger individuals have a lower smoking prevalence (20), residual confounding might have influenced the observed age effect on TRAb dynamics. However, the strong and consistent association between younger age and faster TRAb decline suggests that smoking alone does not explain the age effect.

Younger patients were significantly more likely to reach TRAb levels <10 IU/L earlier after surgery. The stratified analysis by age (Table 2) further confirmed that younger patients achieve TRAb normalization more rapidly, which is consistent with the results of our multivariate analysis identifying younger age as a favourable predictor. Several clinical and immunological mechanisms may explain this trend. Older age is often a proxy for longer disease duration, and prolonged autoimmunity promotes the establishment of long-lived plasma cells in the bone marrow, which can sustain antibody production independent of the target antigen (21). In older patients with a presumably longer history of GD, these autoreactive plasma cells may accumulate over time. Consequently, even after total thyroidectomy removes the primary antigenic source, TRAb production by these extrathyroidal long-lived plasma cells may persist, delaying immunological remission compared to younger patients with shorter disease duration. It is also important to consider the potential influence of GO, as severe GO is more prevalent in older patients and could theoretically contribute to sustained TRAb levels via local antibody production (22). However, in our multivariable analysis, age remained an independent predictor of delayed TRAb decline, even after adjusting for preoperative TRAb levels, which are known to correlate with GO severity. This finding supports the hypothesis that delayed clearance in older patients is driven primarily by systemic immunosenescence, specifically the accumulation and prolonged survival of bone marrow-resident plasma cells, rather than by local orbital pathology alone. Undergoing thyroidectomy at a younger age, before long-standing autoimmune damage or fibrosis develops, may facilitate faster resolution of autoimmunity. As an IgG1 antibody, TRAb is cleared via systemic immunoglobulin metabolism, and younger individuals may have more active reticuloendothelial function, enhancing antibody clearance (16). These factors may act together to accelerate TRAb decline in younger patients.

Although age-related TRAb kinetics is not well documented in surgical series, our findings suggest that younger patients attain immunological remission earlier after thyroidectomy. Previous studies have emphasised TRAb decline before pregnancy to reduce fetal and neonatal risks (10), underscoring the clinical importance of early TRAb control in this population. A notable finding of this study is that younger age was a predictor of earlier TRAb decline, a trend that directly opposes the established evidence in antithyroid drug (ATD) therapy, where younger patients typically exhibit higher relapse rates and slower TRAb decline (23). This contrast highlights the distinct immunological advantages of surgery in younger individuals.

We propose a new paradigm for therapeutic decision-making: unlike ATD therapy, where youth is a risk factor for resistance (9), total thyroidectomy may be most immunologically effective when performed early in the disease course, specifically in younger patients, before the permanent establishment of long-lived plasma cells in the bone marrow. Thus, surgery should be considered not merely as a last resort for ATD failure but also as a strategic option to achieve timely immunological remission in this specific population.

Our findings indicate that total thyroidectomy may be a particularly powerful option for achieving timely immunological remission in this younger patient population.

### Clinical implications for pregnancy

Mothers with active GD are typically managed with ATDs, which affect both maternal and fetal thyroid functions (24, 25). However, persistently elevated TRAb levels during pregnancy pose significant risks, including fetal and neonatal hyperthyroidism (5, 6, 13, 24). Given that TRAb levels exceeding approximately 10 IU/L are strongly predictive of neonatal thyrotoxicosis (13, 14), timely immunological remission is crucial for women planning pregnancy to minimise fetal transfer and subsequent risk. This aligns with the ETA recommendations, which underscore TRAb monitoring and advocate definitive treatment in selected women of childbearing age to minimise fetal risk (9). In our cohort, >80% of the patients reached TRAb levels <10 IU/L within 5 years of surgery, with the most significant reductions occurring in the first year. Although ATDs remain the first-line therapy for women of childbearing age, our findings support total thyroidectomy in selected cases, in which early TRAb decline is desired. In this context, we used 40 years as a pragmatic cut-off to distinguish the typical childbearing-age group, who were most likely to seek early TRAb decline for pregnancy planning, from older patients. Notably, international surveys indicate that thyroidectomy use for GD has increased in recent years (26). Surgery is generally avoided during pregnancy and reserved for exceptional cases that require multidisciplinary management and precise timing (27). Compared with medical therapy, thyroidectomy may offer faster and more consistent TRAb reduction, potentially reducing the fetal risk in appropriately selected patients.

### Potential relevance to Graves’ orbitopathy

Multiple studies have demonstrated that serum TRAb levels are strongly correlated with the clinical activity and severity of GO (22, 28). Therefore, the rapid and sustained decline in TRAb levels observed after total thyroidectomy is biologically plausible as a contributor to the remission or stabilisation of GO. Indeed, unlike radioactive iodine therapy, which can cause a transient increase in TRAb and potentially exacerbate GO (29), total thyroidectomy avoids this risk and is considered immunologically safer for patients with active eye disease.

However, whether this immunological advantage translates into superior clinical outcomes remains debatable. A recent meta-analysis confirmed that total thyroidectomy leads to greater reductions in TRAb than other modalities, but found that this did not consistently result in statistically significant differences in clinical GO improvement (30). The 2016 EUGOGO/ETA guidelines similarly emphasise that the management of GO should be individualised and not solely based on TRAb levels (29). In our cohort, standardised GO activity/severity data were unavailable; therefore, we did not analyse GO outcomes and refrained from making definitive therapeutic claims.

### Limitations

This study had some limitations. First, this was a retrospective analysis conducted at a single high-volume endocrine surgery centre. Despite the large sample size and standardised surgical techniques, selection bias may limit generalisability. Second, although the timing and frequency of post-operative TRAb monitoring were not strictly uniform, most patients in our institutional practice return approximately every 6 months after total thyroidectomy. Thus, the variability in sampling intervals is minimal, and the impact on time-to-event analyses is likely negligible. Third, smoking status was self-reported at baseline with no post-operative monitoring; thus, peri-operative abstinence or relapse could not be verified, and any null association with smoking should be interpreted with caution. As younger patients have lower smoking rates, residual confounding could partly influence the observed age effect on TRAb dynamics. Fourth, data on thyroid-stimulating antibody (TSAb), a functional subset of TRAb with greater specificity for predicting fetal hyperthyroidism and the clinical course of GO, were unavailable. We measured TRAb serially, but the absence of TSAb data limits the direct assessment of the relationship between functional antibody activity and extrathyroidal outcomes, such as fetal risk or GO severity. Fifth, detailed clinical data on GO severity or activity (e.g. clinical activity score) were not systematically recorded for all patients. This prevented us from directly including GO status as a covariate in the multivariate analysis, although we adjusted for pre-operative TRAb levels, which correlate with GO severity. Sixth, the assay’s upper detection limit of 400 IU/L reduces the precision in patients with extremely high pre-operative TRAb levels. True baseline values may have exceeded this threshold, potentially leading to underestimation of the initial decline. Seventh, due to the nature of our institution as a specialised referral centre, detailed data on disease duration, longitudinal trends in thyroid function tests (fT3/fT4) and cumulative ATD dosage before referral were not available for the entire cohort. Many patients have been treated elsewhere for varying periods before surgery, making it difficult to accurately determine the precise onset of the disease or its clinical severity at initial diagnosis. Therefore, we could not include disease duration as a variable or adjust for these potential confounders in our multivariate analysis. The observed association between older age and delayed TRAb decline may partially reflect the impact of prolonged disease duration and the associated accumulation of long-lived plasma cells, rather than age alone. Eighth, although age was a significant predictor of TRAb level reduction, the underlying biological mechanisms remain unclear. Further research involving immunophenotyping or cytokine profiling may elucidate age-related differences in TRAb clearance after surgery. Finally, although TRAb trends were analysed relative to various clinical factors, their impact on long-term outcomes, such as recurrent hyperthyroidism, GO progression, or patient-reported quality of life, was not evaluated. Prospective studies are needed to validate TRAb decline as a surrogate marker of immunological remission.

## Conclusion

In this study, we demonstrated sustained TRAb decline following total thyroidectomy for GD. Most patients reached TRAb levels <10 IU/L within 3 years post-surgery. Earlier remission was linked to younger age, lower pre-operative TRAb level, and lower thyroid weight. Age, previously considered a minor factor, has emerged as a significant determinant of TRAb kinetics, suggesting a potential immunological advantage of surgery in younger individuals. These findings support total thyroidectomy as a strategic option for achieving early TRAb decline, especially in patients planning pregnancy or those with GO who require prompt immunological remission.

## Supplementary materials



## Declaration of interest

The authors declare that there is no conflict of interest that could be perceived as prejudicing the impartiality of the work reported.

## Funding

This work did not receive any specific grant from any funding agency in the public, commercial, or not-for-profit sector.

## Author contribution statement

TS: conceptualization (lead); methodology (lead); formal analysis (lead); writing – original draft (lead); writing – review and editing (equal); case collection (equal). MK: methodology (equal); supervision (lead); writing – review and editing (equal); case collection (equal). MF: data curation (lead); writing – review and editing (equal); case collection (equal). HM: writing – review and editing (equal); case collection (equal). TH: writing – review and editing (equal); case collection (equal). YI: writing – review and editing (equal); case collection (equal). NO: writing – review and editing (equal); case collection (equal). AM: writing – review and editing (equal); case collection (equal). AM: supervision (equal); writing – review and editing (equal); case collection (equal). TA: writing – review and editing (equal); endocrinological guidance (lead).
