# Supplementary material for: Clinical predictors of TRAb decline after total thyroidectomy in patients with Graves’ disease
Source: Eur Thyroid J. 2026 Jan 7;15(1):ETJ250284. doi: 10.1530/ETJ-25-0284 (PMC13156668; doi:10.1530/ETJ-25-0284)
Supplement: Supplementary file 1 [file supplementary_materials.pdf]

**Supplementary Figure 1. Median and Mean Serum TRAb Levels Before and After Total Thyroidectomy.**

Longitudinal trends in serum TRAb levels (n = 1,516). Both the median (solid line) and mean (dashed line) values declined progressively at each postoperative time point, demonstrating sustained TRAb decline after total thyroidectomy.

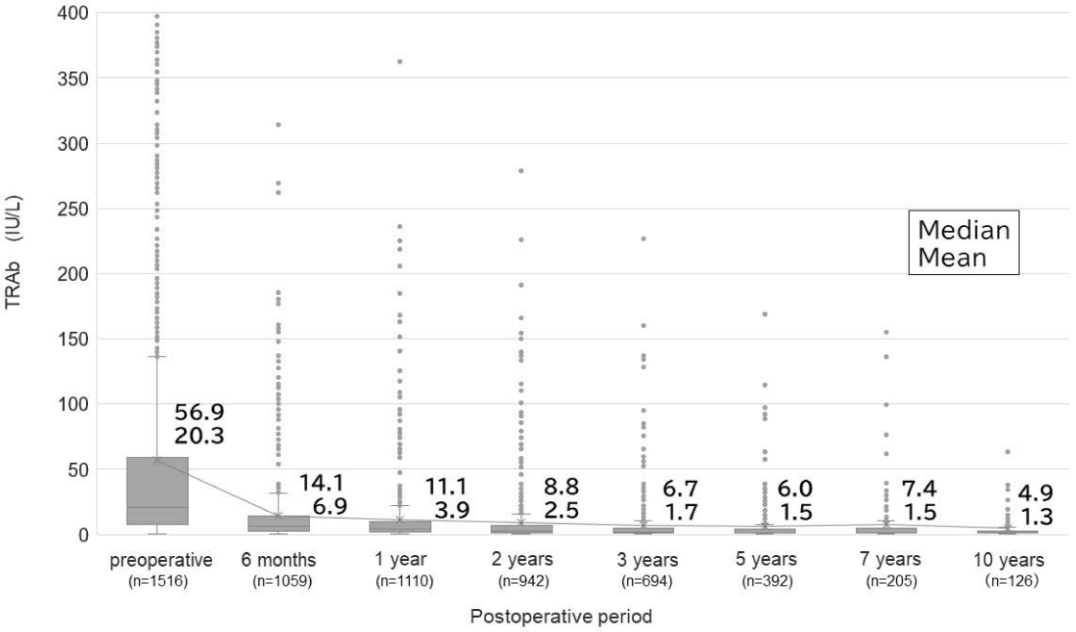

**Supplementary Figure 2. Correlation Between Preoperative TRAb Levels and Resected Thyroid Weight.**

The scatter plot shows the correlation between the preoperative TRAb levels and resected thyroid weight. A significant positive correlation was observed (Spearman's ρ = 0.35, $P < 0.001$), indicating that higher TRAb levels were associated with greater thyroid weight.

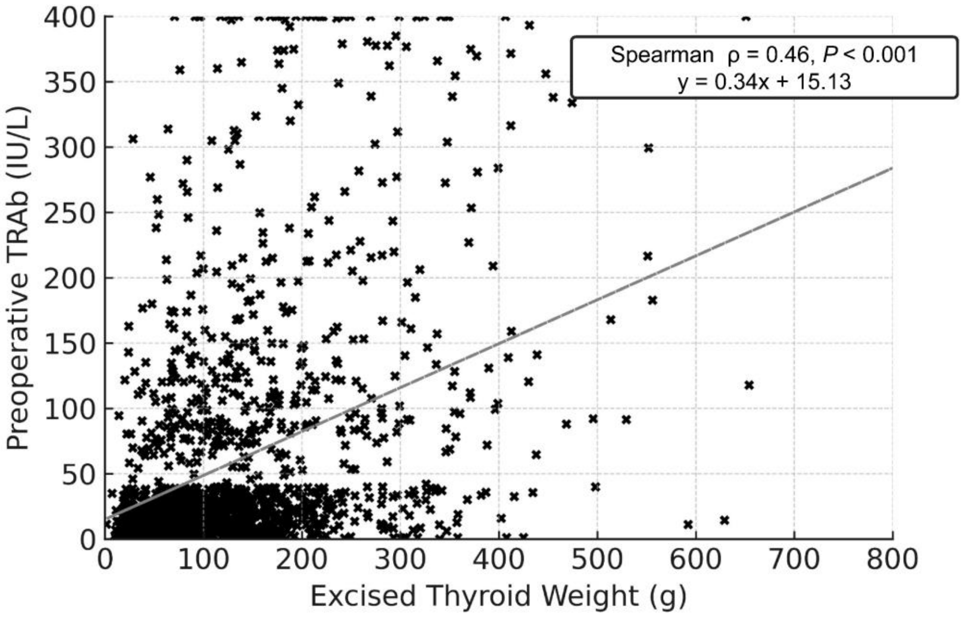

**Supplemental Table 1a. Multivariable Logistic Regression Analysis Identifying Predictors of Reaching TRAb Level <10 IU/L Within 1 Year After Total Thyroidectomy (Continuous Variables)**

| Variable | OR | 95% CI | *P* value |
|---|---|---|---|
| Age (years) | 0.96 | 0.93–0.99 | 0.008 |
| BMI (kg/m$^2$) | 1.01 | 0.99–1.02 | 0.310 |
| Preoperative TRAb (IU/L) | 0.92 | 0.90–0.95 | <0.001 |
| Weight of Resected Thyroid (g) | 0.95 | 0.92–0.97 | <0.001 |

This table presents the odds ratios (OR), 95% confidence intervals (CI), and P values for clinical variables associated with reaching TRAb level <10 IU/L within 1 year after surgery. A TRAb threshold of 10 IU/L was used as a surrogate marker for clinical immunological remission. Age, BMI, preoperative TRAb levels, and weight of resected thyroid were analysed as continuous variables. An OR <1.00 indicates that a higher value of the variable is associated with a lower likelihood of reaching TRAb level <10 IU/L within 1 year.

**Supplemental Table 1b. Multivariable Logistic Regression Analysis Identifying Predictors of Reaching TRAb Level <10 IU/L Within 3 Years After Total Thyroidectomy (Continuous Variables)**

| Variable | OR | 95% CI | *P* value |
|---|---|---|---|
| Age (years) | 0.97 | 0.94–0.99 | 0.017 |
| BMI (kg/m$^2$) | 1.01 | 0.99–1.02 | 0.296 |
| Preoperative TRAb (IU/L) | 0.95 | 0.93–0.97 | <0.001 |
| Weight of Resected Thyroid (g) | 0.96 | 0.93–0.99 | 0.013 |

This table presents the odds ratios (OR), 95% confidence intervals (CI), and P values for clinical variables associated with reaching TRAb level <10 IU/L within 3 years after surgery. A TRAb threshold of 10 IU/L was used as a surrogate marker for clinical immunological remission. Age, BMI, preoperative TRAb levels, and weight of resected thyroid were analysed as continuous variables. An OR <1.00 indicates that a higher value of the variable is associated with a lower likelihood of reaching TRAb level <10 IU/L within 3 years.
